# Reviews and Book Notices

**Published:** 1881-02

**Authors:** 


### Reviews and Book Notices.

ARTICLE XII.—HEALTH AND HEALTHY HOMES: A GUIDE TO DOMESTIC HYGIENE. By George Wilson, M.A., M.D., Medical Officer of Health for Mid-Warwickshire Sanitary District, and author of "Handbook of Hygiene and Sanitary Science." With notes and additions by J. G. Richardson, M.D., Professor of Hygiene in the University of Pennsylvania; 12mo., pp. 314, cloth, $1.50. Philadelphia: Presley Blakiston; 1880.

Considering its size, this is a well-digested and complete treatise of hygiene. It is tersely written, contains none but the most accurate views and facts related to diseases, and will prove a great aid to the public in general, and to medical students especially. Several important points are well established in this work: the certainty of the prevention of diseases through sanitary measures; the prolongation of human life, as a necessary result of the same; and the extinction of some dreadful epidemic diseases which used to decimate the human race almost every decade, some centuries ago.

The physiology of the human body; the causes of disease, among which hereditary transmission and causes induced by the individual himself, and sanitary precautions, are all treated in a scientific manner. But the reader will soon perceive that this is the *résumé* of a larger work, and devoid of anecdotes and pleasant digressions, which would make its perusal less fatiguing. The conclusion terminates with this peculiar remark:

"Then, too, it has to be pointed out that so long as medical practitioners are paid solely for their services in attending on cases of illness, it is manifestly not to their interest, from a purely monetary point of view, to assist in the prevention of disease, because if prevention means anything at all, it certainly implies that the more effectually the causes of disease are removed, and precautionary measures are carried out, the less numerous ought patients to become, and the sick-rate and death-rate will both be lowered. It is true that medical men, with the noble disinterestedness which characterizes the profession, are seldom lax in doing all they can to prevent the spread of disease; but there is no disguising the fact that in many quarters there is appearing on the surface a growing antagonism between medical practitioners and sanitary authorities, which is very much to be regretted. All this might be obviated if the public could be persuaded that it would be to their ultimate advantage to pay the medical attendant a liberal allowance to conserve the health of the household."

The reviewer thinks the remark offensive for honest physicians. The antagonism referred to exists, but depends on personal contentions, places secured through political influences, the election of ignorant or over-anxious officers, and other causes, rather than on the desire to legally murder. H. D. V.

ARTICLE XIII.—THE PRACTITIONER'S HANDBOOK OF TREATMENT, OR PRINCIPLES OF THERAPEUTICS. By J. Milner Fothergill, Member of the Royal College of Physicians of London; Assistant Physician to the City of London Hospital for Diseases of the Chest, and to the West London Hospital, etc., etc. Second American, from the second London edition, enlarged; pp. 641.

The favorable reception of two large editions of this work in England and America, proves its acceptability to the profession. Its aim is to supply a digest of the general principles of therapeutics, to arrange the well-known facts of practice together with the explanations furnished by pathological research and physiological inquiry, in such form that the treatment of each individual case becomes an intelligent and rational procedure. This difficult task the writer accomplishes in admirable style, though no attempt is made to collect all the information possible or to enumerate all the articles of the pharmacopœia. The work comprises twenty-four chapters in which the diseases peculiar to each physiological system are discussed as to pathology and the appropriate therapeutical treatment suggested.

The contents of the seventh chapter, on Growth and Decay; of the twelfth, on Diatheses and Cachexia; of the fifteenth, on the Respiratory System; the twenty-second, on Public and Private Hygiene, are especially worthy of mention and the concluding twenty-fourth chapter, entitled The Medical Man at the Bedside, in which the author quotes largely from Prof. Austin Flint's "Clinical Medicine," will be of special value to the young practitioner, and its careful perusal will save his incautious steps from many a humiliating stumble.

The book is valuable in that it is suggestive and stimulates the reader's own therapeutical knowledge, and though an occasional view of the author will be found at variance with those of the majority of American practitioners; as for instance, the administration of atropia in doses that seem dangerously large, and the advice not to withhold it when the atropine phenomena appear, which we fancy few patients outside of hospital wards will allow. Still, pathology as morbid physiology is the strong point of the work, and an occasional therapeutical fault does not detract much from its value, as it is not designed to supplant the regular text-books on materia medica and therapeutics, but as a valuable adjuvant. D.

ARTICLE XIV.—A MANUAL OF MINOR SURGERY AND BANDAGING. By Christopher Heath, F.R.C.S., Surgeon to University College Hospital, and Holme Professor of Clinical Surgery in University College, London; Honorary Fellow of King's College. Sixth edition. Revised and enlarged; with 115 illustrations; 12mo., pp. 342, cloth, $1.50. Philadelphia: Lindsay & Blakiston; 1880.

This book contains all that is necessary for the diagnosis and treatment of surgical diseases coming under the care of house-surgeons, and we may say of the average practitioner. It contains also many hints not found in larger works, comparative tables of symptoms, various manners of treatment, and all the latest discoveries and improvements of real value in mechanical surgery. The fact that it has already reached its sixth edition renders any praise superfluous, yet let it be said that it is the best book published on the subject, and one whose perusal will repay the student with interest.

Nothing has been forgotten, every suggestion is useful and practical, and the style is orderly, easy and interesting. The author has identified himself with the subject. The illustrations are neat wood-cuts, handsomely executed, which will render the text easy even for beginners. H. D. V.

ARTICLE XV.—DIAGNOSIS AND TREATMENT OF EAR DISEASES. By Albert H. Buck, M.D., Aural Surgeon to the New York Eye and Ear Infirmary; Instructor in Otology in the College of Physicians and Surgeons of the City of New York, etc.

This work forms an admirable addition to the list of American medical literature. With but a half dozen lines of preface in which the author announces that his aim has been to present the results of his own experience in text-book form, he addresses himself at once to his subject.

Chapter I gives the physiology of the ear in simple, lucid style, illustrated by some satisfactory diagrams of the mechanical arrangement of the ossicles and of the minute anatomy of the middle ear, vestibule, semicircular canals and cochlea. Chapter II is devoted to the examination of patients, tests of the hearing power, details of instruments and methods. He strongly deprecates the use of the syringe, even by beginners, in the art of otological manipulation, recommending the curette instead, supplemented by the probes and cotton holder. The idea, however, of the country practitioner, with perhaps a half dozen otological cases a year, removing impacted ceramus from the meatus with the curette, will hardly be enthusiastically received by the great majority of the profession. Still the syringe is recommended for the patient's use, and the style represented has its point well protected from damages to the membrane by projecting bars. Chapter III, on Diseases of the Auricle. IV, of the External Auditory Canal. V, Methods of Examining the Middle Ear. VI and VII, Diseases of the Middle Ear, purulent and non-purulent. Chapter VIII is devoted to Fractures of the Temporal Bone, with a series of selected cases, fourteen in number, a subject not usually treated in works of this kind, and forms a valuable section. Cnapter IX contains seventy-four pages on Diseases of the Mastoid Process, with five good illustrations of the anatomy of the mastoid region.

There are forty-six illustrative cases, detailed, with their clinical history and treatment. Chapter X is devoted to Miscellaneous Conditions of the Drum Membrane, Ossicles and Tympanic Cavity, of which want of space forbids detail, but interesting. Chapter XI on different forms of disease, in which the Labyrinth is supposed to be involved closes the book, except a convenient index.

This book is distinctively American, fresh and original in arrangement and detail. It deserves better type and binding than that in which this volume appears. Wm. Wood & Co.'s Cheap Series, of 1880. D.

ARTICLE XVI.—A TEXT BOOK OF PHYSIOLOGY. By M. Foster, M.A., M.D., F.R.S., Prælector in Physiology and Fellow of Trinity College, Cambridge. With Illustrations. Third Edition, Revised, 8vo. Cloth, pp. 720. $3.50. London: MacMillan & Co., 1879.

This book is well adapted for students, on account of the amount of information it contains, of the regular arrangement of the matters treated, and of its cheapness, which is worth some consideration in reference to class books; but it will not replace the classical works of Dalton and of Flint, Jr.

The essentials of physiology are all contained in that volume, some in rather a concise manner, as Menstruation, in less than three pages, Impregnation, two pages, Nutrition of the Embryo, six, and Parturition, two. In an appendix of fifty-eight pages, small type, is treated the Chemical Basis of the Animal Body, a study which is fast increasing in the medical world, and is destined, some suppose, to revolutionize in the near future, our treatment of diseases.

The Chapters on Vascular Mechanism, the Contractile Tissues, Digestion, Respiration and Nutrition, form the bulk of the volume. It may be remarked that the fluidity of normal blood, the origin of the red corpuscles, the question, Do we see objects upside down, and the origin of the special nerve of taste are yet unsatisfactorily explained? Regarding the beat of the heart, the author says: "From these facts (p. 169), the conclusion is drawn that the spontaneous pulsations in the heart are in some way associated with, and due to the action of the ganglia scattered in its substance."

The style of printing and binding is inferior to that of medical books in general, at least in this country. It is well illustrated, and will command a large sale on account of its medium size. The publishers announce a cheap "Student's" Edition, but the "Text Book" is surely not too voluminous for American medical students. H. D. V.

ARTICLE XVII.—HEADACHES: THEIR NATURE, CAUSES AND TREATMENT. By W. H. Day, M.D., Member of the Royal College of Physicians of London; Physician to the Samaritan Hospital for Women and Children. Third edition, with illustrations. 12mo. Cloth, pp. 322. Price $2.00. Philadelphia: Lindsay & Blakiston. 1880.

The first page, on opening the book, being devoted to Opinions of the Press, it would be difficult to surpass the praises there addressed to this deserving little book. It is surely a fine example of modern book making, and will greatly profit the buyer, if he can afford to pay $2.00 for such a monograph. It is all original matter, and the treatment, which is the best part of the book, is methodical and adapted to all sorts of cases and circumstances.

It is handsomely bound and beautifully printed, contains 116 valuable and practical formulæ, and a chapter devoted to Headaches of Childhood and Early Life. H. D. V.

